# Why Patients Delay Their First Contact with Health Services After Stroke? A Qualitative Focus Group-Based Study

**DOI:** 10.1371/journal.pone.0156933

**Published:** 2016-06-08

**Authors:** Alice Le Bonniec, Julie Haesebaert, Laurent Derex, Sylvie Porthault, Marie Préau, Anne-Marie Schott

**Affiliations:** 1 Hospices Civils de Lyon, Pôle Information Médicale Evaluation Recherche, Lyon, France; 2 Laboratoire Epsylon EA4556, Université Paul Valéry Montpellier, Montpellier, France; 3 Groupe de Recherche en Psychologie Sociale (GRePS) EA4163, Université Lumière Lyon 2, Lyon, France; 4 Hospices Civils de Lyon, Unité de neurologie vasculaire, Hôpital Pierre Wertheimer, Lyon, France; 5 SAMU du Rhône, Hospices Civils de Lyon, 69000, Lyon, France; 6 Institut National de la Santé et de la Recherche Médicale U912 (SESSTIM), Marseille, France; 7 Universite de Lyon, Equipe d’Accueil HESPER 7425, F-69003, Lyon, France; Cardiff University, UNITED KINGDOM

## Abstract

**Background:**

Despite national and local French information campaigns, when acute stroke occurs, waiting times before calling mobile emergency medical services (EMS) to receive appropriate treatment (i.e. thrombolysis) and decrease the risk of physical disability, remain long. We aimed to identify the representations of stroke in the general population and to determine barriers to and facilitators for rapidly contacting EMS.

**Method:**

We conducted a qualitative study among the general population with 10 focus groups, 5 comprising employed people (N = 29) and 5 comprising retirees (N = 32). The themes discussed were general knowledge about stroke and its risk factors, symptoms, appropriate management and the awareness that stroke is an emergency issue.

**Results:**

In addition to a lack of knowledge about stroke, other barriers to rapidly contacting the EMS were difficulties in recognizing symptoms and understanding that these symptoms constitute an emergency. Furthermore, when faced with stroke, a feeling of inevitability and fatalism about the consequences of a stroke was highlighted. Participants were unaware of the existence of an effective treatment and they mistrusted medical competences. Finally, we found a strong presence and participant appreciation of common knowledge, resulting in the sharing of experiences of stroke. This could partly compensate for the lack of specific knowledge about symptom recognition and appropriate action.

**Conclusion:**

Information campaigns should not only inform the public about stroke symptoms in order to ensure people act appropriately, but should also focus on increasing public awareness about the fact that an effective treatment exists.

## Introduction

How sufferers and witnesses react in the immediate moments after an acute stroke influences access times to stroke units and consequently thrombolysis treatment, which in turn influences functional prognosis [1]. In order to administer thrombolysis within four and a half hours after a stroke—after a CT scan or an MRI in a specialized stroke unit (SU)–initial actions to help the patient must be appropriate and prompt [2]. Time in stroke management can be divided into two phases: pre- and in-hospital management. In France, as in other western countries, many interventions have been implemented to improve stroke care through a national action plan, mainly at the in-hospital phase. However, the rate of thrombolysis treatment remains suboptimal, in part because of extended pre-hospital delays [3,4].

Existing literature shows that when sufferers and witnesses are presented with a situation of acute stroke, they typically delay their reaction, and either do not call EMS immediately or do not call at all. This prevents patients from accessing optimal treatment [5]. A systematic review by Jones *et al*. revealed that the ability of the public to name symptoms was generally poor [6]. Two other reviews concluded that most stroke campaigns were ineffective [7] and lacked any theoretical framework for their development [8].

To design more effective campaigns, a greater understanding of the general population’s representations and beliefs about stroke is essential, as these are determinants of behaviors. We performed a qualitative study to identify representations of the general population about stroke and determine barriers to and facilitators of rapid calls to EMS when a possible stroke occurs.

## Materials and Methods

This qualitative study was undertaken with focus groups using a phenomenological approach. A total of 10 focus group discussions took place with individuals from the general population, 5 groups comprising retirees and 5 comprising employed participants. We aimed to identify similarities and differences between the perceptions of these two groups.

### Focus groups

The focus group approach was chosen to induce interaction between participants and to observe the co-construction of representations, and the sharing of experiences and common knowledge between participants [9]. Using focus groups pushes participants not only to take a stance and explain their individual points of view, but also to adapt themselves to the standards created by the group. It is also the most suitable method for studying social representations [9]. The focus groups created here were moderated by ALB; JH and NB were observers. The groups consisted of semi-structured discussions, which followed an interview grid consisting of 5 themes based on available stroke prevention literature (presented in Table 1). For each theme, written, audio or visual support helped to initiate discussions. Participants were invited to discuss each theme freely. A pilot session was performed among professionals from our unit who are not involved in stroke research or in qualitative studies and members of their family. The interview grid was modified in light of results from this pilot session. Focus groups took place in a meeting room at our research unit in Lyon.

**Table 1:** Themes developed in focus groups.

**Theme 1**	Knowledge about stroke: word association task (participants were asked to write the first three words that came to mind when the word "stroke" was mentioned)
**Theme 2**	Stroke risk factors and sense of vulnerability (testimonial video of a victim of stroke*)
**Theme 3**	Symptoms of stroke and stroke recognition (participants were presented the results of a study which investigated the proportion of persons who reported theoretically knowing what the symptoms of stroke are)
**Theme 4**	Management of stroke: most appropriate actions to take when faced with a stroke, where and how to manage stroke patients (proposals of various reactions and ranking by participants)
**Theme 5**	The sense of emergency: which diseases and accidents can be considered as warranting an emergency response? (reading article extracts about emergency unit attendance and overcrowding)

* The testimony video showed a stroke survivor who related her experience. The video was selected because it was neutral enough to not influence participants’ responses.

### Recruitment–Sample

We recruited a convenience sample of employed participants and retirees from among members of the French mutual health insurance group APICIL in Lyon. We randomly contacted 2,000 members matching inclusion criteria (1,000 employed and 1,000 retirees) by mail, inviting them to participate. All met the following inclusion criteria: aged between 30 and 65 years for employed and between 65 and 80 years for retirees, and living in the district of our research unit (in order to facilitate venue to focus groups). A total of 307 favorable responses (116 employed and 191 retirees) were received by phone, mail or e-mail (global response rate 15%; employed 12%; retirees 20%). It was not possible to know the reasons for refusal to participate. With the respondents who agreed to participate, we created 5 groups of employed participants and 5 groups of retirees, each comprising up to 9 participants and having a sex ratio of 1:1. Sixty-one participants were finally included to compose the 10 focus groups. Written informed consent was obtained from all participants. The study was approved by the national French data protection authority (CNIL) and an institutional review board (CPP SUD ESTII).

### Analysis

All focus group meetings were audio- and video-recorded and notes were taken by observers during meetings. Data saturation was reached after 5 focus group in each population (i.e. each of the 10 focus groups met once), therefore, no additional focus group had to be created. ALB transcribed the interviews verbatim and performed a thematic content analysis using the interview grid and NVIVO software (NVivo QSR International). The content analysis followed steps defined by Bardin [10]. Vertical and transversal analyses of interviews were performed to categorize the whole verbatim transcripts into themes and subthemes. This type of analysis helps to highlight opinions of the majority of participants and select the most representative quotes. The word association task (i.e. the first three words cited by the participants at the mention of the word "stroke") was analyzed separately. We investigated the influence of common knowledge in the construction of representations in greater depth. Common knowledge represents knowledge that is based on past self-experiences, as opposed to knowledge based on scientific sources [11]. As an example, common knowledge on stroke management is based on personal experience, or the experience of someone in one’s social or working circle, while scientific knowledge is based on medical practices and scientific guidelines on stroke management.

## Results

### Study population characteristics

Ten focus group discussions took place, involving 61 participants (mean of 6 participants per group; minimum 3; maximum 9). Interviews lasted on average 1h32min (1h18–1h42). Characteristics of the 61 participants are presented in Table 2. Forty-six participants knew someone in their social or work circle who had suffered from a stroke, and 3 participants themselves had already suffered from a stroke.

**Table 2:** Characteristics of study participants (N(%)).

		Retirees: N = 32	Employed: N = 29	Total: N = 61
**Gender**	Male	17 (53%)	9 (31%)	26 (43%)
	Female	15 (47%)	20 (69%)	35 (57%)
**Family situation**	Married / couple	18 (56%)	21 (72%)	39 (64%)
	Single	4 (13%)	5 (17%)	9 (15%)
	Separated/ Divorced	4 (13%)	3 (10%)	7 (11%)
	Widowed	6 (19%)	0 (0%)	6 (10%)
**Personal history of stroke**		1 (3%)	2 (7%)	3 (5%)

### Knowledge of stroke—Words association analysis

Words quoted by the participants were grouped by theme. The most common words cited reflected dependence (e.g., "*paralysis*", "*disability*"). Discrepancies were highlighted between employed individuals and retirees, the former more often mentioning words referring to the unpredictability of stroke (such as "*sudden*", "*attack*", "*unexpected*") and the fatal character of stroke (such as "*dead*", "*death*", "*impotence*"). Retired participants more frequently mentioned more positive topics such as “*prevention*”, “*explanation*” and “*action*”.

### Thematic analysis

Table 3 summarizes the main results of the thematic analysis.

**Table 3:** Summary of the main results based on each theme discussed.

**Definition and risk factors of stroke: sense of vulnerability**	Inaccurate definition of stroke
	Factors associated with a feeling of vulnerability: presence of risk factors, knowing a stroke victim, the unpredictability of stroke which can strike everyone
**Stroke onset: symptoms of stroke and stroke recognition**	Lack of knowledge about symptoms
	Variability and / or non-specific symptoms resulting in difficulty recognizing stroke
**Calling EMS and sense of emergency**	Symptoms which are not very alarming
	Symptoms which do not reflect the severity of the situation (no pain no vital distress)
	Emergency department overcrowding
**Consequences of stroke—Management of stroke**	No knowledge of stroke treatment possibilities
	Lack of confidence in hospital care
	Strong sense of fatalism

EMS: emergency medical service

#### Definition and risk factors of stroke: sense of vulnerability

All participants spontaneously declared that they did not know anything about stroke and expressed a need to deepen their knowledge about its causes and to question their own vulnerability.

Stroke seemed a vague concept to most participants. They wondered about its origin, symptoms and treatment. Indeed, the definition of stroke which they had understood was not always clear to them and gave rise to questions, especially regarding the difference between ischemic and hemorrhagic stroke.

*"In my point of view*, *it's both*, *it's just the clot that makes the blood vessel explode"* (R31G8, retired male)

*"Is “Stroke” the scientific word for brain aneurysm? I know this is a blood vessel that explodes but not necessarily in the brain*, *it can be anywhere in the body"* (A23G9, employed woman)

With respect to risk factors, participants raised concerns about the unpredictability of stroke and the fact that there is no standard profile for a stroke sufferer. For a majority of participants it can “*strike everyone*”. In all focus groups, stroke was qualified by its unpredictability, and the term "sword of Damocles" emerged in three groups. This term refers to something which is out of one’s reach and control, and which decides a person’s fate. Only patients who had cardiovascular risk factors reported them as stroke risk factors. The sense of vulnerability varied according to age and some of those employed considered that only the elderly are concerned by stroke.

"*No*, *for me*, *stroke was for the elderly*. *I thought it was attrition*, *I thought it affected only the elderly*." (A3G1, employed woman)

#### Stroke onset: symptoms of stroke and stroke recognition

In all focus groups, when questioned about how to recognize a stroke, the majority of participants initially declared they were not familiar with any symptoms of stroke. Then, as the discussion progressed, when remembering their own or their relatives’ experiences of stroke, participants started to cite some symptoms. Paralysis and aphasia were the most cited symptoms. The main barrier to contacting EMS was the lack of knowledge of symptoms. *"The problem is that it is not in both directions*: *if it is a stroke*, *there's one of these symptoms*, *but if there's one of these symptoms there isn’t necessarily a stroke; so that's why they may be reluctant to contact the EMS*. *But on the other hand it is a precaution*.*"* (R3G3, retired male).

The seriousness of stroke symptoms and their consequences not being perceived as dangerous, led individuals to minimize the emergency.

*"I would say it would bother me to call the EMS for the symptoms mentioned*, *because I have the feeling that*…*it is being a bit alarmist*, *a deformation of the mouth*…*of speech*, *it may be due to something else*, *I do not know*, *these elements are not very meaningful for me*.*"* (R2G3, retired male).

#### Calling EMS and sense of emergency

The feeling that the situation was not serious enough to require the intervention of EMS and the fear of being blamed for disturbing was present in 5 focus groups.

"*It is true what A2 says*, *when we visit our GP*, *either for our children or for us*, *he always says “wait three days*, *if symptoms persist come back to me”*. *So it is with this intention of not overloading emergency units and not increasing the burden on the national health insurance system*…"(A1G1, employed woman). This refers to social conformity pressure. The problem of emergency departments and overcrowding led to participants feeling guilty about contacting the EMS.

#### Consequences of stroke and stroke management

Most participants associated negative outcomes with stroke, such as physical disability and wheelchair use. However, participants had no knowledge of acute stroke treatment options. No participant was familiar with thrombolysis. The feeling of inevitability and powerlessness constituted an important barrier to calling EMS.

(An account by one participant of stroke prognosis for two of his relatives who were treated 50 years apart for a stroke): *finally, when I think about what happened and then what I knew later, I've known people who have had a stroke for over 50 years now…and in those 50 years, there’s been no change [in care]. Either you become invalid or you die… and finally, often the preferred solution is to die”*. (R13G6, retired man)

Some participants also expressed a lack of confidence in the medical care system, which is also something that may influence people’s behavior regarding whether to call the EMS or not.

*"For myself*, *in the case of [A8G1*, *employed woman]*, *what greatly shocked me*, *is that the medical community was very slow at diagnosing stroke*. *So it's not very reassuring at all*.*”* (A3G1, employed woman)

### Transversal analysis—Common knowledge

The experience of stroke, whether direct or indirect (i.e., happening to oneself, or to relatives or acquaintances), was strongly present in the groups discussions. Participants used common knowledge to try to determine the symptoms of stroke and the most suitable actions to take, to assess the likelihood of stroke happening to themselves, and finally, to try to predict the consequences. The narrative of the experience of relatives with stroke initiated many discussions and led to several interactions between participants. For example, one retired participant responded to another who asked if speech disorder should alert one’s attention to the possibility of stroke:

*"Yes*, *I have an aunt who died of a stroke*, *she had several strokes in fact*, *and the first time the sign was that she could no longer talk on the phone*, *she could no longer speak*.*"* (R14G6, retired woman)

In contrast, those who did not know any stroke victim and who had never experienced stroke did not feel that it was legitimate to speak and give their point of view. This would suggest that in a real-life situation such people might be reluctant to act promptly (i.e. contact EMS) because of their lack of previous stroke experience and knowledge.

*"Yes*, *there is nobody I know around me who had a stroke*, *so obviously I cannot talk about it*.*"* (R16G6, retired male)

Common knowledge could be more important than scientific knowledge for individuals. For example, some participants questioned the symptoms presented in the stroke sensitization posters presented at the end of the focus groups, as the symptoms they had experienced (directly or indirectly) were different. The words of one retired participant reading stroke symptoms presented on a poster illustrated this challenge to scientific knowledge:

*"Well then I didn’t see any deformation of the mouth*, *I didn’t see any weakness on one side of the body*. *Speech disorder*, *well*, *there was no speech anymore*, *so it is not a “disorder”*…*"* (R12G5, retired woman)

## Discussion

We studied representations of stroke in the general population and barriers to calling EMS when stroke occurs. A lack of knowledge about stroke symptoms, and considering that stroke symptoms do not warrant emergency action, were two factors highlighted by both employed participants and retirees. The former more frequently referred to the unpredictability and inevitability of stroke, which can strike anyone without warning, while the latter were more focused on stroke prognosis and recovery. For both populations, representations of stroke were mainly built on personal and relatives’ experience. The main barriers to contacting EMS when confronted with a stroke were a lack of knowledge, a sense of incapacity to identify stroke symptoms and to understand the urgency of the situation, a fear of unnecessarily increasing the burden on emergency services, and a sense of powerlessness. The latter was true for all the stakeholders involved. Participants had no idea that an effective treatment (i.e., thrombolysis) is available and that it must be quickly administered.

The lack of knowledge about stroke symptoms and the difficulty in recognizing them have already been reported as the largest barriers to calling EMS [6]. Interestingly, participants knew that a stroke was an emergency, but did not connect the symptoms to stroke or to an emergency. These results are consistent with those from other studies [12,13]. The variety of acute stroke symptoms, which do not always correspond to symptoms presented in campaigns, was another difficulty reported in our study and others [12].

The unpredictability of stroke was of great concern to participants [14], a strong sense of fatalism being reported. Participants also associated poor prognosis with stroke, characterized by the evocation of death and physical disability in the word association task. This is consistent with other studies [12,14]. More unexpected was a lack of confidence in the medical care system which constituted a barrier to contacting EMS. This emerged from several of the 10 focus groups. Some participants reported the experience of relatives who had had sub-optimal management leading to long intra-hospital delays. Moreover, participants’ lack of knowledge about effective treatment (i.e., thrombolysis) reinforced the sense of fatalism, and this may in turn have induced a sense that urgent reaction is useless.

Another obstacle to contacting EMS was the fear of annoying medical care providers and of unnecessarily congesting emergency services. This result is in line with that of Mackintosh who showed that the first call made was often for a relative, in order to obtain confirmation that the situation required a physician. Additionally, like Dombrowski, we found that witnesses were more likely to call EMS than stroke sufferers themselves [15]. This could be attributable to the cognitive impact of stroke leading to a denial of symptoms and anosognosia, and/or to social conformity. The denial of symptoms and the guilt associated with calling EMS may lead to the decision to wait to see if symptoms disappear [12].

Stroke, probably because of its brutal and unpredictable characteristics, does not correspond to the usual social representation ascribed to it. Indeed, a link can be made with the work of Sarradon -Eck [16], who demonstrated that high blood pressure (a risk factor for stroke) has the image of being a "silent disease" and " sneaky ". This is confirmed by the peculiarity of stroke, whose symptoms do not seem all that alarming and may be difficult to recognize because symptoms vary from one person to another, and thus "hide" its seriousness. Our study has limitations. First, it was performed on a convenience voluntary-based sample in Lyon, with a high proportion of participants knowing victims of stroke. Three of the sample were themselves stroke survivors. Participants were probably more educated and more sensitized to health and stroke than the general population. Emphasizing the importance of common knowledge, opinions of the three stroke survivors were particularly valued in the groups. Indeed, these stroke survivors and witnesses of stroke were considered by others to have expert knowledge about the matter. In light of this, we ensured that speaking was equitably distributed during the focus group meetings, to avoid the possibility that those with greater first-hand knowledge would dominate the discussion. Although the convenience nature of the sample could represent a bias, a convenience sample was employed to meet the requirement of having a diversified population for the focus groups. Stroke survivors contributed to this diversity. Another limitation is that our study did not include young participants, who could have different representations about stroke, and are also a target for stroke information campaigns.

The lack of scientific knowledge leads people to turn to another form of knowledge: common knowledge. Our results are consistent with those of Derex *et al*. and show that knowing someone who experienced a stroke was an independent factor of increased ability to recognize stroke symptoms [3].

The sense of powerlessness felt by participants faced with stroke (happening to themselves or to someone else) may represent a barrier to primary prevention strategies. Indeed, stroke is viewed by participants as unpredictable and inevitable. This point is of crucial interest when designing future educational campaigns.

Our study may provide some clues for improving communication about stroke. To counteract the feeling of fatalism, campaigns should indicate that an effective treatment exists if delivered in time. This could increase patient empowerment and knowledge about efficacy of prevention and therapeutic strategies, and their adherence to treatments. Posters are meaningless for the public and ineffective for improving the recognition of stroke symptoms. Instead campaigns should rely more on videos showing symptom characteristics. Knowledge is gained by sharing experiences. This important fact should lead to campaigns being built around a vicarious model [17]. Accordingly, campaigns should not only act on knowledge, but also on behavior, explaining how to act when stroke occurs and describing the benefit of this action. For example, a campaign showing a person recognizing symptoms of a stroke, immediately calling EMS, and showing the improvement of the prognosis in the future could increase the likelihood that stroke witnesses react the same way in real-world situations. Interventions should be designed to empower populations, increase self-efficacy, and emphasize the benefits of acute stroke treatment. A survey on stroke preparedness in the US and the UK showed that stroke recognition and response were improved with greater belief that medical treatment can help and better understanding of stroke [18]. Another study in UK showed that an increase in knowledge about stroke had an impact on emergency department attendance [19]. It must be noted nonetheless that efficacy in shortening delays remains limited [20] and that the efficacy of mass media campaigns has to be interpreted with caution, because the links between knowledge and behavior, and between behavioral intention and actual behavior are not certain [21].

## Conclusion

Our study provides information on how individuals construct their thinking patterns when faced with an acute stroke. This information could help improve the efficacy of acute stroke sensitization campaigns. Key issues to emphasize in future stroke awareness campaigns—in addition to stroke symptoms—are that everyone has the power to act, that there is a benefit from acting rapidly, and that an effective medical treatment for stroke exists.
